# Emergency obstetric referrals in public health facilities: A descriptive study from urban Maharashtra, India

**DOI:** 10.3389/frhs.2023.1168277

**Published:** 2023-04-17

**Authors:** Sushmita Das, Sarita Patil, Sweety Pathak, Sahana Chakravarthy, Armida Fernandez, Shanti Pantvaidya, Anuja Jayaraman

**Affiliations:** Society for Nutrition, Education and Health Action, Mumbai, India

**Keywords:** maternal health, emergency obstetric care, referral system, Mumbai, India

## Abstract

**Background:**

An effective referral system is key to access timely emergency obstetric care. The criticality of referrals makes it necessary to understand its pattern at the health system level. This study aims to document the patterns and primary reasons of obstetric case referral and the maternal and perinatal outcome of the cases in public health institutions in select areas of urban Maharashtra, India.

**Methods:**

The study is based on the health records of public health facilities in Mumbai and its adjoining three municipal corporations. The information on pregnant women referred for obstetric emergencies was collected from patient referral forms of municipal maternity homes and peripheral health facilities between 2016 and 2019. Maternal and child outcome data was obtained from “Received-In” peripheral and tertiary health facilities to track whether the referred woman reached the referral facility for delivery. Descriptive statistics were used to analyze demographic details, referral patterns, reasons of referrals, referral communication and documentation, time and mode of transfer and delivery outcomes.

**Results:**

14% (28,020) women were referred to higher health facilities. The most common reasons for referral were pregnancy-induced hypertension or eclampsia (17%), previous caesarean section (12%), fetal distress (11%) and Oligohydramnios (11%). 19% of all referrals were entirely due to unavailability of human resources or health infrastructure. Non-availability of emergency Operation Theatre (47%) and Neonatal Intensive Care Unit (45%) were the major non-medical reasons for referrals. Absence of health personnel such as anaesthetist (24%), paediatrician (22%), physician (20%) or obstetrician (12%) was another non-medical reason for referrals. Referring facility had a phone-based communication about the referral with the receiving facility in less than half of the cases (47%). 60% of the referred women could be tracked in higher health facilities. Of the tracked cases, 45% women delivered *via* caesarean section. Most of the deliveries (96%) resulted in live birth outcomes. 34% of the newborns weighed less than 2,500 grams.

**Conclusion:**

Improving referral processes are critical to enhance the overall performance of emergency obstetric care. Our findings emphasize the need for a formal communication and feedback system between referring and receiving facilities. Simultaneously, ensuring EmOC at different levels of health facilities by upgradation of health infrastructure is recommended.

## Introduction

Significant progress has been made globally in saving mothers' lives in the last two decades. The number of maternal deaths has declined from 451,000 to 295,000 between 2000 and 2017; a reduction of 38 percent worldwide (1). In spite of this remarkable progress, nearly 810 women died globally from preventable causes related to pregnancy and childbirth every day in 2017 (1). Maternal mortality is a key indicator of maternal health and is indicative of the performance of a country's health care system (2). Most maternal deaths are due to direct obstetric causes and can largely be prevented with access to appropriate health care including presence of skilled birth attendance at delivery and timely referrals to emergency obstetric care services (3–5).

India's maternal mortality ratio (MMR) has witnessed a sharp decline from 398 per 100,000 live births in 1997–98 to 103 per 100,000 live births in 2017–18 (6, 7). This decline can be attributed to the considerable efforts that have been made to strengthen the health infrastructure as well as to improve access to skilled birth attendants at delivery (8, 9). Despite these efforts, there is a lot of ground to be covered to attain the Sustainable Development Goal of MMR below 70 by 2030 (10).

Maternal death audits from India imply multiple referrals as one of the major reasons for maternal deaths (11). World Health Organization (WHO) defines referral as “a process in which a health worker at one level of the healthcare system, having insufficient resources (drugs, equipment, and skills) to manage a clinical condition, seeks the assistance of a better or differently resourced facility at the same or higher level to assist in or take over the management of, the client”s case” (12). Ensuring accurate and timely risk screening in antenatal period to prevent or treat the high-risk condition under specialized care is the principle of a formalized maternal referral system (13). In addition, a well-defined protocol for referrals ensures optimal use of the hospital services and timely care of patients at the appropriate level.

**Figure 1:**
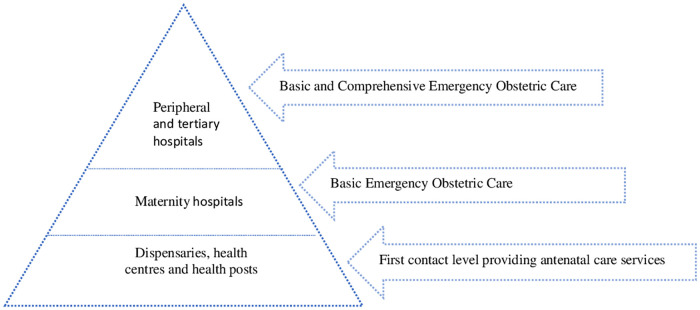
Urban public health system for obstetric care in India.

India's public health system is pyramidal in structure and aims to improve service delivery while reducing workload at the tertiary level and strengthening the peripheral infrastructure (14). As indicated in Figure 1, in urban areas, dispensaries, health centres and health posts are the primary level of contact for antenatal care. Delivery care services are provided by maternity hospitals. Emergency obstetric care (EmOC), a package of services to treat obstetric complications, is expected to be provided by maternity hospitals and the aforementioned primary level of contact. EmOC consists of two components; Basic Emergency Obstetric Care (BEmOC) and Comprehensive Emergency Obstetric Care (CEmOC) (15). Maternity hospitals, the next level in the pyramidal structure, have been proposed as Basic Emergency Obstetric Care centres. Peripheral and tertiary hospitals are supposed to provide both basic and comprehensive EmOC (16, 17).

In spite of a well-defined referral system, lack of basic essential care at referring facilities, inadequate information exchange between referring sites, and direct referrals from primary to tertiary level facilities contributing to overcrowding at the apex facilities are some of the factors leading to poor referrals in India (13, 18–20).

The urban referral system is complex compared to the rural system. While there may be a concentration of health infrastructure in urban areas, public health systems in large cities are often challenged by inequitable spatial distribution of health facilities, unsuitable distances from urban informal settlements, weak referral systems with sub optimally utilized primary care institutions and overloaded tertiary hospitals (17, 21). Studies on provincial and national referral systems in different countries have identified similar challenges around accessing care, which includes inadequate resources and infrastructure at lower levels, underutilization of secondary level hospitals, patients' preference to access higher level health facilities for basic care, overload of the apex hospital with referrals, and an inadequate health information system for patient referral (22–25).

Society for Nutrition, Education and Health Action (SNEHA) had partnered with public health systems in the Mumbai Metropolitan Region to initiate a formal maternity referral network among health facilities to strengthen the referral mechanisms for maternal and newborn health in the urban context. A review conducted by SNEHA in Brihanmumbai Municipal Corporation (BMC) prior to the initiation of the project revealed that unavailability of various facilities such as Operation Theatre, provision for laboratory investigations and around the clock availability of medical staff were the major reasons for referrals. To address the gaps in the referral system, the project was initiated in 2004 as a pilot in two regions (Central and Eastern) of BMC and was subsequently extended to include the Western region in 2008. The purpose of the project was to implement a provider participatory model for empowering health care personnel to initiate and strengthen referral processes among the facilities and to help mothers receive the most appropriate care. As a substantial number of the emergency high-risk cases were referred to BMC from adjoining corporations, strengthening of the referral processes in these corporations was a pressing priority. With the purpose, in 2012, the project was scaled up to these three additional corporations of Mumbai Metropolitan Region to establish regional referral linkages between primary, peripheral and tertiary care centres within the municipality, and adjacent corporations. Presently, SNEHA partners with these municipal corporations to strengthen and sustain these linkages.

There is limited evidence on the maternal referral system across urban India. A review of current literature available for urban India suggests that most of the studies were conducted in tertiary healthcare facilities where the referral cases were received (13, 14, 26–28). Our study drew on secondary data from referral hospitals across all levels of public health facilities from four municipal corporations. This descriptive study aims to document the patterns and primary reasons of obstetric case referrals and the maternal and perinatal outcome of the cases in public health institutions.

## Materials and methods

### Study design

The study is part of implementation research to establish a robust referral system for high-risk pregnant women for improved maternal and neonatal outcomes. Retrospective research design was used for the study. The data was collected from municipal hospital records across 33 maternity, 16 peripheral and 4 tertiary hospitals between 2016 and 2019. The study population consisted of obstetric referrals from municipal maternity (Level I) and peripheral (Level II) hospitals.

### Study setting

The Mumbai Metropolitan Region is spread over 6,328 sq. km. and consists of 9 Municipal Corporations with a population of over 26 million (29). It is among the most populous metropolitan areas in the world. This study was conducted in four corporations in the Region which are largely urban.

Brihanmumbai Municipal Corporation: The city is densely populated across 603.4 sq. km with a population of 12,442,373 of which 42% resides in slums (26). The Corporation's Department of Public Health administers tertiary medical colleges, specialist hospitals, peripheral general hospitals, maternity hospitals, dispensaries, and health posts. Within a broad range of programmes, these provide preventive, promotive and curative services for mothers and children.

Thane Municipal Corporation: The city is adjacent to Mumbai, the financial capital of India, covering an area of 147 sq. km and a population of 1,841,488. Slums consist of 18% of the total population (26). Within the Public Health Department of the Municipal Corporation, there are Urban Primary Health Centres (UPHCs) which offer outpatient department services, maternity hospitals, a general hospital and a medical college. Complicated cases are sometimes referred to hospitals within the BMC.

Mira Bhyander Municipal Corporation: The city is located about 53 kilometres northeast of Mumbai. The total population is estimated at 814,655 of which 8% resides in slums (26). Health services are provided through general hospitals, UPHCs and mobile dispensaries. Complicated cases are referred to BMC when necessary.

Kalyan Dombivli Municipal Corporation: It is a suburb of Mumbai, located to the North with an area of 79.4 sq. km and a population of 1247,327. The total slum population is 52,318 (26). The Public Health Department includes ten UPHCs and two general hospitals. Tertiary care health facility is not available within the corporation and high-risk cases are referred to BMC for treatment.

### Data collection

To facilitate effective communication and implementation between referred and referral facilities, a standardized referral documentation format called referral slip was developed by SNEHA in collaboration with the Municipal Corporations (MCs). The slip contained demographic information of the referred women (name, age, address and contact number), referral information (time of referral, reason for referral, medical conditions, place of referral, and any related information), obstetrics information (gravida, parity, number of live births, number of abortions and any other investigations conducted), treatment information at the time of referral, any pre-referral communication, and mode of referral. Prototype of the referral slip remained consistent across all health facilities to ensure that the relevant information was provided whenever a referral was initiated. Referral slips were provided to all facilities in the referral chain, along with training on appropriate referral documentation for safe and effective transfer of women. The booklet of slips was used for referral documentation. The original was given to the woman and the carbon copy was kept in the facility for records. The information from the duplicate slips for women referred from municipal maternity homes and peripheral health facilities were collected by a team of SNEHA investigators every month. Investigators also collected information on “Received-In” data from peripheral and tertiary (Level III) health facilities. Information collected from referral slips were matched with “Received-In” data to track whether the referred woman reached the referral facility for delivery. Data collected from the receiving facilities included referral details (reason for referral, place of referral), admission details (date and time) and delivery information (mode of delivery, time of delivery) and outcome (neonatal outcome/baby weight/condition/APGAR score if available). Data on number of deliveries and their type and time of referral, among others, were also collected from Level I, II and III facilities as part of the implementation process. During the initial phase of the project, doctors from different levels of care came together to develop clinical protocols, which defined the obstetric conditions that were to be managed at each level and those that were to be referred to higher levels (peripheral and tertiary) of facilities. The protocols were reviewed and approved by a committee comprising of senior obstetricians from academic departments of public tertiary centers and other leading members of professional bodies of the same discipline. This protocol was used to review appropriateness of referrals for 10% of the referred cases with complete documentation. The criteria such as mother's general and obstetric condition, foetus's status, capacity of the facilities in the linkage, and the immediate outcome of the mother and the newborn at delivery were studied by an experienced obstetrician to assess whether the woman had an appropriate referral.

Inclusion criteria for the study was referrals of pregnant women with gestational weeks of 28 weeks and above. Referral cases with less than 28 weeks of gestation and abortion were excluded as they usually do not have neonatal outcomes.

Data was entered on smartphones using CommCare (https://www.dimagi.com/commcare/), an open-source software application running on the Google Android operating system (www.android.com). The database management system included built-in skip patterns, acceptable ranges, and constraints to ensure a reduction in errors associated with data collection and data entry. Data were routinely checked after downloading for errors in key fields.

### Statistical analysis

Data was analysed using STATA V.14. Referrals, normal deliveries and caesarean sections and tracking were described as proportions. Referral proportion was defined as the number of referrals divided by the total number of referrals and deliveries conducted in a particular facility. Completeness of the referral slips was captured by the number of fields filled in the slip. The slip had ten major fields (name and age of the woman, index pregnancy details, reason for referral, medical condition at the time of referral, provisional diagnosis, details of clinical investigations conducted, date and time of referral and name of the facility referred to). A referral slip was considered complete if all ten fields were filled. Tracking was defined as the number of cases tracked over referrals. Normal Delivery/Caesarean section was the number of births delivered by vaginal birth/Caesarean section divided by the total number of deliveries (any delivery method). Intrauterine Fetal Deaths (IUFDs) occurring ≤23 weeks' gestation were recorded as IUFDs, whereas those ≥24 weeks' gestation were categorized as stillbirths. Newborns weighing less than 2,500 grams were considered as Low Birth Weight (LBW) babies. Descriptive statistics were used to analyse demographic details like age, gravida, referral patterns, medical and non-medical reasons of referrals, referral communication and documentation, time and mode of transfer and delivery outcomes.

## Results

**Figure 2:**
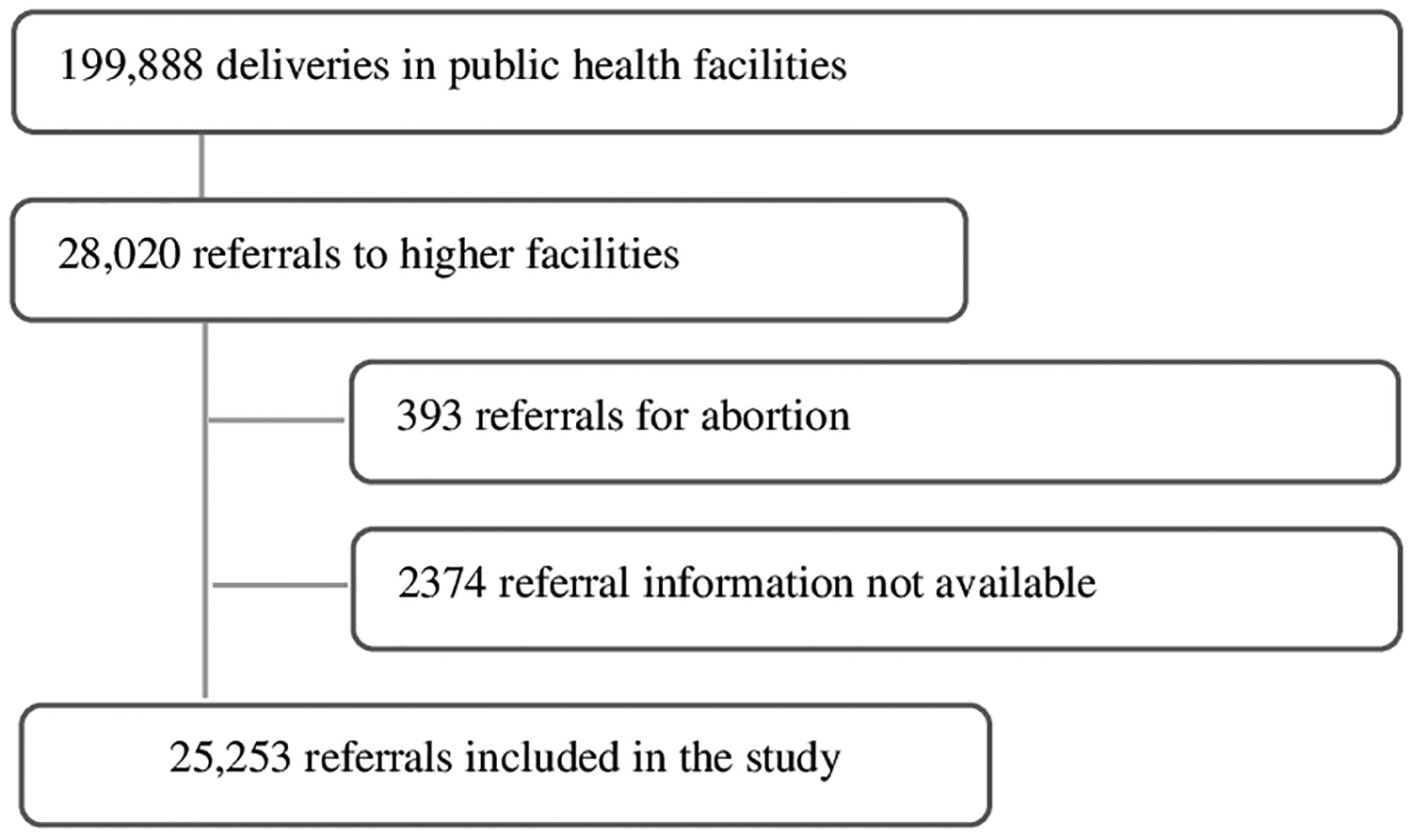
Study flow chart.

Figure 2 shows the study flow chart. Between 2016 and 2019, a total of 199,888 deliveries were conducted in Level I and II of public health facilities in four municipal corporations with 28,020 (14%) referrals to higher health facilities for obstetric emergency care. Of the referred cases, referrals for abortion services were excluded from the analysis (393) and 2,374 cases were not included in the analysis as complete information on the place of referral was not available. The final sample included 25,253 women.

Table 1 summarizes the profile of the referred women. Majority (74%) of them were less than 30 years of age. 14% were aged 30 years and above. Mean age of the women was 24 (SD 6) years. Nearly half of the women (46%) were primigravid and a similar proportion of women (46%) were multi or grand multigravida.

**Table 1:** Profile of women referred to higher level of public health facilities across four municipal corporations in urban Maharashtra, India, 2016–2019.

Woman’s age (in years)	*n* (%)
18–23	9,039 (36%)
24–29	9,654 (38%)
30+	3,572 (14%)
Missing	2,988 (12%)
Mean (SD)	24 (6)
Gravida	
Primigravid	11,510 (46%)
Multigravida	11,267 (45%)
Grand multigravida	380 (1%)
Missing	2,096 (8%)

**Figure 3:**
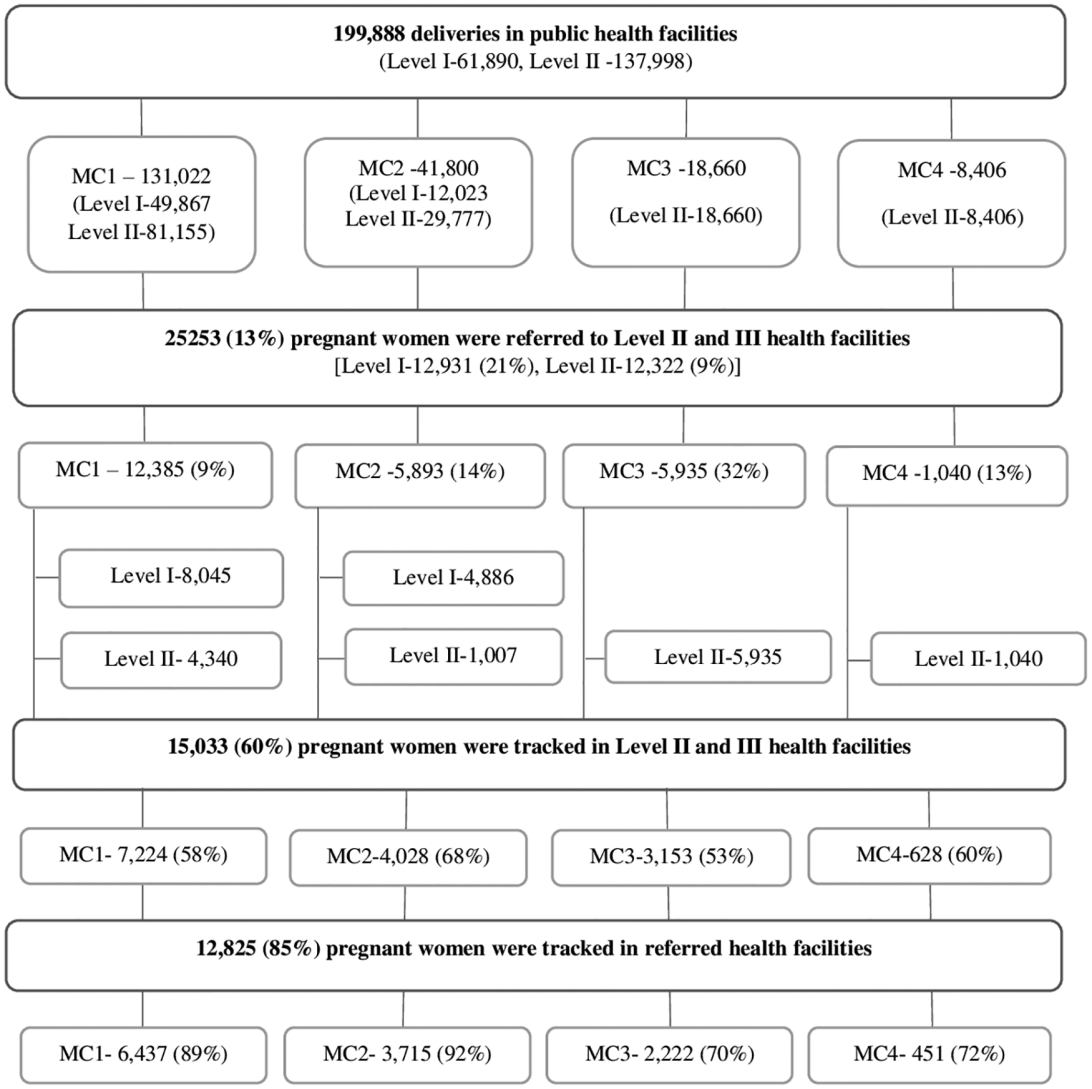
Referrals and tracking in public health facilities across four municipal corporations in urban Maharashtra, India, 2016–2019.

Figure 3 summarizes the referral pattern and tracking status of pregnant women by corporation. Pseudo names MC 1, MC 2, MC 3 and MC 4 were used to describe the results to retain the anonymity and confidentiality of the corporations.

During the study period, 199,888 pregnant women accessed Level I and II health facilities for childbirth. Of these women, 61,890 (31%) and 137,998 (69%) accessed Level I and Level II health facilities, respectively. In MC 3 and 4, all admissions were in Level II health facilities. Of all the women accessing Level I and II health facilities, 25,253 (13%) were referred to higher health facilities. Referrals were higher from Level I (12,931, 21%) as compared to that of the Level II health facilities (12,322, 9%). There were variations in referrals across the corporations. Referrals were high in MC 3 (32%), followed by MC 2 (14%), MC 4 (13%) and MC 1 (9%). Referrals from Level I health facilities was considerably high in MC 2. In MC 3 and 4, all referrals were from Level II health facilities as Level I health facilities were not functional during the study period. Referrals were inter-corporation in both the MCs due to unavailability of tertiary health facilities within the corporations whereas in MC 1 and 2, all referrals were intra-corporation.

Of all the referrals, we could track 15,033 (60%) women in higher health facilities of which 12,825 (85%) women reached the designated referral facility. This compliance was high in MC 2 with 92% of women reaching the appropriate referred destination. A similar proportion of women adhered to the referral advice and reached the selected referral facility in MC 1 (89%) but adherence was lower for MC 3 (70%) and MC 4 (72%).

Table 2 summarizes the obstetric causes of referral. Obstetric causes were grouped into ante- and intra-partum causes and each of the groups included maternal and fetal indications for referrals. Often women had multiple complications at the time of referral, hence the primary cause of referral could not be determined in most cases. The most common reasons for referral were pregnancy-induced hypertension or eclampsia (17%), previous caesarean section (12%), fetal distress (11%) and oligohydramnios (11%). Data suggests that in 24% of cases, referrals were exclusively for obstetric reasons. Corporation-wise breakdown of data shows that in MC 2, more than half of the cases (54%) were referred exclusively for obstetric causes, followed by MC 1 (18%), MC 4 (10%) and MC 3 (9%).

**Table 2:** Diagnosis at the time of referral in public health facilities across four municipal corporations in urban Maharashtra, India, 2016–2019.

A. Antepartum	MC 1 (*N* = 9,184)	MC 2 (*N* = 4,692)	MC 3 (*N* = 3,263)	MC 4 (*N* = 873)	Total (*N* = 18,012)
*Maternal indications*	*n* (%)	*n* (%)	*n* (%)	*n* (%)	*n* (%)
Previous caesarean section	954 (10%)	521 (11%)	602 (18%)	139 (16%)	2,216 (12%)
Abnormal presentation of the baby	593 (6%)	314 (7%)	220 (7%)	74 (8%)	1,201 (7%)
Cephalopelvic Disproportion (CPD)	619 (7%)	328 (7%)	519 (16%)	41 (5%)	1,507 (8%)
Non progress of labour	496 (5%)	346 (7%)	121 (4%)	43 (5%)	1,006 (6%)
***Fetal indications***					
Fetal distress	1,279 (14%)	458 (10%)	153 (5%)	79 (9%)	1,969 (11%)
Preterm labour	851 (9%)	259 (6%)	431 (13%)	94 (11%)	1,635 (9%)
Preterm premature rupture of membranes	1,082 (12%)	273 (6%)	249 (8%)	31 (4%)	1,635 (9%)
Intrauterine growth restriction (IUGR)	579 (6%)	147 (3%)	292 (9%)	81 (9%)	1,099 (6%)
Intrauterine fetal death (IUFD)	324 (4%)	56 (1%)	48 (1%)	25 (3%)	453 (3%)
**B. Intrapartum**					
***Maternal indications***					
Bad obstetric history	59 (<1%)	35 (<1%)	18 (<1%)	4 (<1%)	116 (<1%)
Pregnancy induced hypertension/Eclampsia	1,436 (16%)	853 (18%)	591 (18%)	114 (13%)	2,994 (17%)
Medical disorders (anaemia, jaundice, malaria, asthma, diabetes)	1,084 (12%)	261 (6%)	329 (10%)	162 (19%)	1,836 (10%)
***Fetal indications***					
Fetal anomaly	118 (1%)	32 (<1%)	23 (<1%)	17 (2%)	190 (1%)
Multiple gestation	132 (1%)	61 (1%)	58 (2%)	14 (2%)	265 (1%)
Oligohydramnios	1,009 (11%)	497 (11%)	376 (12%)	138 (16%)	2,020 (11%)
Polyhydramnios	92 (1%)	94 (2%)	69 (2%)	20 (2%)	275 (2%)
Referrals exclusively due to obstetric reasons	2,045 (18%)	2,713 (54%)	470 (9%)	99 (10%)	5,327 (24%)

Obstetric causes of referral were not available for 7,241 (29%) women.

Table 3 presents the non-medical reasons for referrals. In majority of the cases, more than one reason was stated for referral with significant overlap between human resources and infrastructure-related reasons. Non-availability of emergency Operation Theatre (47%) and Neonatal Intensive Care Unit (NICU) (45%) were the major reasons for referrals to higher facilities. Variations were observed across municipal corporations in referrals for emergency operation theatres with 52% in MC 2 as compared to that of 28% in MC 4. Similar variations were observed for NICU referrals (62% in MC 3 vs. 35% in MC 1). Referrals for Medical Intensive Care Unit (MICU) were high in MC 3 (38%) and MC 4 (29%). Other major reasons for referrals were unavailability of health personnel such as anaesthetist (24%), paediatrician (22%), physician (20%) and obstetrician (12%). Requirements of blood bank (14%) and clinical investigations (10%) were also documented as reasons for referral. Data suggests that in 19% of cases, women were referred only for non-medical reasons. Obstetric reasons were not mentioned in the referral slips in these cases. MC 3 had the highest proportion (36%) of such referrals, followed by MC 1 (18%), MC 4 (13%) and MC 2 (6%).

**Table 3:** Non-medical reasons for referral as recorded in referral slips in public health facilities across four municipal corporations in urban maharashtra, India, 2016–2019.

Non-medical reasons	MC 1 (*N* = 9,198)	MC 2 (*N* = 2,304)	MC 3 (*N* = 4,624)	MC 4 (*N* = 908)	Total (*N* = 17,034)
	*n* (%)	*n* (%)	*n* (%)	*n* (%)	*n* (%)
Emergency OT required	4,600 (50%)	1,194 (52%)	1,872 (40%)	255 (28%)	7,921 (47%)
NICU required	3,186 (35%)	1,300 (56%)	2,857 (62%)	391 (43%)	7,734 (45%)
MICU required	909 (10%)	69 (3%)	1,740 (38%)	260 (29%)	2,978 (17%)
Anaesthetist not available	2,348 (26%)	96 (4%)	1,403 (30%)	215 (24%)	4,062 (24%)
Paediatrician not available	2,490 (27%)	484 (21%)	778 (17%)	28 (3%)	3,780 (22%)
Physician not available	1,155 (13%)	242 (11%)	1,816 (39%)	129 (14%)	3,342 (20%)
Obstetrician not available	883 (10%)	113 (5%)	928 (20%)	74 (8%)	1,998 (12%)
Blood bank required	1,183 (13%)	137 (6%)	1,041 (23%)	39 (4%)	2,400 (14%)
Clinical investigations	717 (8%)	75 (3%)	912 (20%)	79 (9%)	1,783 (10%)
Others	633 (7%)	182 (8%)	107 (2%)	30 (3%)	952 (6%)
Referrals exclusively due to non-medical reasons	2,059 (18%)	325 (6%)	1,831 (36%)	134 (13%)	4,349 (19%)

Non-medical reasons for referral was not available for 8,219 (33%) women.

Table 4 suggests that in less than half of the cases (47%) referring facility had a phone-based communication about the referral with the receiving facility. Variation across the corporations can be seen as 25% of the referrals were communicated to the receiving facility in MC 3, followed by MC 1 (51%), MC 4 (56%) and MC 2 (62%). Completion rates of referral slips was low at 37%. Nearly one third of the slips were filled in MC 3 (32%) and MC 2 (29%) as compared to 43% and 41% in MC 1 and MC 4, respectively. Analysis of the appropriateness of referred cases indicated that 78% of the completely documented cases were appropriately referred *(Not included in Table)*.

**Table 4:** Inter facility referral communication, transfer, timing and documentation in public health facilities across four municipal corporations in urban maharashtra, India, 2016–2019.

	MC1 (*N* = 12,385)	MC2 (*N* = 5,893)	MC3 (*N* = 5,935)	MC4 (*N* = 1,040)	Total (*N* = 25,253)
Referral communication	*n* (%)	*n* (%)	*n* (%)	*n* (%)	*n* (%)
Communication by phone	6,274 (51%)	3,634 (62%)	1,500 (25%)	580 (56%)	11,988 (47%)
No communication	6,111 (49%)	2,259 (38%)	4,435 (75%)	460 (44%)	13,265 (53%)
Complete documentation	5,283 (43%)	1,701 (29%)	1,870 (32%)	425 (41%)	9,279 (37%)
Transferred in ambulance	5,764 (47%)	3,605 (61%)	1,523 (26%)	541 (52%)	11,433 (45%)
Time of referral					
8 am to 8 pm	6,783 (55%)	3,168 (54%)	3,227 (54%)	602 (58%)	13,780 (55%)
8 pm to 8 am	3,771 (30%)	1,846 (31%)	2,236 (38%)	358 (34%)	8,211 (32%)
Not mentioned	1,831 (15%)	879 (15%)	472 (8%)	80 (8%)	3,262 (13%)

Nearly half of the women (45%) were transferred to the higher facility in ambulances. Table 4 also summarizes the time of referral from the referring facility. Referrals were high between 8 am–8 pm (55%) as compared to 8 pm-8 am (32%). This trend was similar across all corporations. Time of referral was not mentioned in the referral slip for 13% of cases (Table 4).

Table 5 shows the delivery and neonatal outcomes. Of all the tracked cases (60%), information on the mode of delivery was available for 93% of cases. More than half of the women (55%) had a vaginal delivery, followed by caesarean section (45%). Caesarean section rates were high in MC 2 (48%) and MC 1 (46%) as compared to the other two corporations. Neonatal outcomes were available for 90% of the tracked cases. Most of the deliveries (96%) resulted in live birth outcomes. Intrauterine fetal deaths comprised of 4% of all cases. No major variation was observed across the corporations. Information on birth weight was available for 92% of the tracked cases of which 34% weighed less than 2,500 grams.

**Table 5:** Obstetric outcomes of tracked study participants in public health facilities across four municipal corporations in urban Maharashtra, India, 2016–2019.

	MC1	MC2	MC3	MC4	Total
Tracked cases	7,224 (58%)	4,028 (68%)	3,153 (53%)	628 (60%)	15,033 (60%)
Delivery mode	*n* (%)	*n* (%)	*n* (%)	*n* (%)	*n* (%)
Vaginal delivery	3,791 (54%)	1,858 (52%)	1,625 (60%)	338 (57%)	7,612 (55%)
Caesarean section	3,281 (46%)	1,701 (48%)	1,089 (40%)	258 (43%)	6,329 (45%)
Neonatal outcome					
Live birth	6,645 (95%)	3,359 (97%)	2,436 (98%)	514 (94%)	12,954 (96%)
Intrauterine fetal death (IUFD)	325 (5%)	101 (3%)	54 (2%)	30 (5%)	510 (4%)
Neonatal deaths	2 (<1%)	–	1 (<1%)	1 (<1%)	4 (<1%)
Still birth	7 (<1%)	–	1 (<1%)	1 (<1%)	9 (<1%)
Birth weight					
Low birth weight	2,671 (38%)	908 (26%)	881 (34%)	226 (38%)	4,686 (34%)

## Discussion

Integration of primary, secondary and tertiary levels of health care is key to better service delivery. An efficient referral system facilitates this integration by linking different levels of care for optimal utilization of health services. There is paucity of studies exploring referral systems in urban settings in India. Our study fills the gap by documenting emergency obstetric referrals in public health facilities in urban areas of Maharashtra.

Our study revealed that overall obstetric referrals were low (13%), though variation in the referral rates based on the level of facilities was observed. The existing public health care system is hierarchical where Level I health facilities are expected to provide the basic Emergency Obstetric Care and provision of comprehensive Emergency Obstetric Care is uncommon. This could be a likely explanation for higher referrals from Level I facilities (21%) as critical cases were required to be referred to higher levels of facilities. Our results show low level of referrals from majority of Level II facilities (9%) and the findings are not in line with recent studies in urban areas that have documented higher rates of referrals (15%–32%) (27–31). One of the probable explanations for the lower referral rates in our study areas could be our work in partnership with the public health system to strengthen the referral processes and improve the coordination among different levels of care so that together, they function as a well-coordinated obstetric service. With this view, the project focused on a participatory approach and ensured representation of providers and administrators of all levels in decision-making. The approach aided in securing buy-in for project processes and contributed to successful establishment of formal linkages. Similar processes were followed to develop referral protocols and guidelines which contributed to their adoption across different levels seamlessly. We do not have comparable reference figures to substantiate our explanations as there was no formal process of referral documentation in the health facilities when the project was initiated.

We could track more than half (60%) of all the referred women reaching the higher facilities for giving birth. A significant number of women were lost to follow-up. The study identified major challenges in following up with women through facility registers. The major reasons included incomplete referral documentation at facility level and error in documentation of demographic details at referral or referred facility.

Compliance is regarded as adherence to the referral advice of the primary level of care facility and reaching the referred destination (32). Other studies have documented low level of compliance as significant number of women do not reach the referred facility (32, 33). Our findings are consistent with these studies as we could not track a substantial number of women (40%). However, of the tracked women, compliance to reach the advised facility was high (85%).

Literature suggests that hypertension or eclampsia, preterm labour, premature rupture of membranes, previous caesarean section, fetal distress and oligohydramnios are the major causes of referral for obstetric care and our findings are in accordance with these studies (21, 28, 34–38). Referrals exclusively due to obstetric causes were low (24%) in our study emphasizing the fact that the majority of the cases could have been managed by the referral facility in case of availability of adequate infrastructure.

In the context of health system, the term “infrastructure” can be used to describe physical infrastructure, manpower (skilled, clinical and supportive staff), drugs, equipment and blood availability which are the prerequisite for health care delivery (39). Our findings suggest that physical infrastructure such as operation theatres, neonatal and maternal intensive care units were not available or non-functional when women needed emergency care. Recent studies have also documented similar reasons for referral (28, 40, 41). A systematic review to identify and categorise specific facility-level barriers to the provision of maternal health care in developing countries cited staff shortages in a health facility as a major barrier to receipt of appropriate care by pregnant women (42). A study conducted in Bangladesh found that human-resource constraints were the major barriers to accessing maternal care (40). An assessment was conducted in Gujarat, one of the states of India to understand the management of EmOC at regional, district and sub-district levels. It was observed that availability of gynaecologist, anaesthetist and obstetrician were inadequate (43). Our findings were in line with these studies and revealed limited availability of trained human resources in health facilities. One-fifth of all referrals in our study were exclusively due to non-medical reasons which suggested that upgradation of health infrastructure can play a critical role in improving maternal and neonatal outcomes.

Referral documentation is an essential component of the referral process. Studies have documented improvements in the quality of referral and care provided at the receiving facility when referral notes are completely documented (44, 45). Audits of referral notes from health facilities have identified gaps in referral documentation that includes lack of essential information such as provisional diagnosis, treatment provided before referral and laboratory investigations (18, 20, 45). Our study reported similar findings where most of the referral slips were not fully documented.

Communication between the referring and receiving facilities is crucial for prompt referral and to allow the receiving facility to prepare for the emergency. Usually, the referral begins with communication from the referring health facility through telephone calls to the receiving facility, based on nature of the obstetric emergency of the pregnant woman. This process is skipped a number of times and referrals are made without any intimation to the receiving facility. This increases the probability of multiple transfers and subject women to further delay and obstetric complications. Studies have demonstrated lack of communication between referral facilities as a major barrier in the referral process and our study is in accordance with these findings (46–48)*.* Limited evidence is available on birth and neonatal outcomes of referred women. Our study fills this gap by following these women through hospital data and documenting the birth outcomes. The World Health Organization recommends an ideal rate of cesarean deliveries between 10% to 15% in any nation (49). This rate cannot be applied as the ideal rate at the hospital level because it varies widely depending on differences in the case-mix of the obstetric populations they serve, their capacity and provisions, and in clinical management protocols (50). This rate increases further in instances of referred women as they have a higher obstetric risk than those women admitted to hospital without referral. A study among referred and self-referred birthing women in Tanzania found caesarean section births in 55% of formally-referred women (51). Our findings are in line with this as nearly half of all women (45%) had caesarean section births. For similar reasons, newborns with low birth weight was higher in our study (34%) as compared to that at the population level (18%) (9). The study revealed that obstetric complications were managed well by the referred facilities as events of adverse neonatal outcomes were low (4%). We did not come across any case of maternal death in our study.

Our findings reiterate the need for referral protocols and guidelines across all levels of health facilities. Nevertheless, given the challenges faced by public health facilities in service provision, such as inadequate infrastructure and human resources, the protocols need to be contextualized in view of the capacity of health facilities to provide services and manage referrals without compromising on the quality of care. Protocols can be updated regularly with changes in the evolving capacity of health facilities to manage cases. Establishing formal referral linkages in consultation with the concerned health facilities may encourage participation in their implementation. Regular interactions among service providers and stakeholders of various levels to discuss referral data, issues and solutions in a participatory manner is important to improve the functioning of the maternity referral network. Gaps in referral documentation and communication can be addressed through regular training and supervision to ensure the adoption and accuracy in documentation of referral slips.

The strengths of the study were its urban settings and its large sample size. One of the main limitations was the research design; this secondary research was not originally designed to collect data for the study. The data was acquired from the public health system records, therefore the researchers did not have control over the content, completeness and accuracy of the data set. Missing data for a few variables was the other limitation that could have contributed to biased results. Nevertheless, the hospital records from 53 public health facilities across four municipal corporations allowed us to access a substantial amount of data. We further theorize that the representativeness of the sample was not affected as the data was missing at random. Lastly, we were unable to follow those referred women who did not access care at the referral centres. Analysing their reasons for dropping out of the referral system would give further insight into the functioning of the network.

## Conclusion

Our findings suggest that the referral rates and adverse maternal and neonatal outcomes were low in our study areas. It reiterates the fact that integration of a referral system and improving processes are critical to enhance the overall performance of emergency obstetric care. Our results emphasize the need for a formal communication and feedback system between referring and receiving facilities. Nonetheless, there is a need to institutionalize these processes into the referral system to sustain improved outcomes. Simultaneously, ensuring EmOC at different levels of health facilities by upgradation of health infrastructure would go a long way in improving maternal and newborn health outcomes.

## Data Availability

The data analyzed in this study is subject to the following licenses/restrictions: All data analysed during this study are available from the corresponding author on reasonable request. Requests to access these datasets should be directed to sushmita@snehamumbai.org.

## References

[1] World Health Organization. Trends in maternal mortality 2000 to 2017: estimates by WHO, UNICEF, UNFPA, World Bank Group and the United Nations Population Division.

[2] Sajedinejad S Majdzadeh R Vedadhir AA Tabatabaei MG Mohammad K. Maternal mortality: a cross-sectional study in global health. Global Health. (2015) 11(1):1–13. 10.1186/s12992-015-0087-y 25889910 PMC4353673

[3] Khan KS Wojdyla D Say L Gülmezoglu AM Van Look PF. WHO Analysis of causes of maternal death: a systematic review. Lancet. (2006) 367(9516):1066–74. 10.1016/S0140-6736(06)68397-9 16581405

[4] Ronsmans C Graham WJ. Lancet maternal survival series steering group. Maternal mortality: who, when, where, and why. Lancet. (2006) 368(9542):1189–200. 10.1016/S0140-6736(06)69380-X 17011946

[5] Essendi H Mills S Fotso JC. Barriers to formal emergency obstetric care services’ utilization. J Urban Health. (2011) 88(2):356. 10.1007/s11524-010-9481-1 PMC3132235 20700769

[6] Meh C Sharma A Ram U Fadel S Correa N Snelgrove JW Trends in maternal mortality in India over two decades in nationally representative surveys. BJOG. (2022) 129(4):550–61. 10.1111/1471-0528.16888 34455679 PMC9292773

[7] Office of the Registrar General I. Special Bulletin on Maternal Mortality In India 2017–19. Sample Registration System. 2022. p. 1–4. Available from: https://censusindia.gov.in

[8] Ministry Of health and family welfare India. Directory of Innovations Implemented in the Health Sector Supported by Department for International Development. (2009).

[9] International Institute for Population Sciences and ICF. National Family Health Survey (NFHS-5), India, 2019–21. (2021). Available from: https://dhsprogram.com/pubs/pdf/FR375/FR375.pdf

[10] World Health Organization. Sustainable Development Goals (SDGs). (2015). Available from: https://sdgs.un.org/goals

[11] Tanzin D Madhu G Manmeet K Sonu G Arun Kumar A Jorge C. Maternal and perinatal death inquiry and response project implementation review in India. J Obst Gynecol India. (2013) 63(2):101–7. 10.1007/s13224-012-0264-3 24431614 PMC3664695

[12] Hort K K Gilbert P Basnayaka PLA. Strategies to strengthen referral from primary care to secondary care in low- and middle-income countries. Vol. 1. World Health Organisation (2019).

[13] Rehman T Keepanasseril A Maurya DK, Kar SS. Factors associated with maternal referral system in South India: a hospitalbased cross-sectional analytical study. J Nat Sci Biol Med. (2020) 11:158–63. 10.4103/jnsbm.JNSBM_33_20

[14] Rekha J Ankita C. Study of maternal outcome in referral obstetric cases in a tertiary care centre. J Family Med Prim Care. (2019) 8:2814–9. 10.4103/jfmpc.jfmpc_402_19 31681648 PMC6820423

[15] Maine D Wardlaw TM Ward VM McCarthy J Birnbaum A Akalin MZ Guidelines for monitoring the availability and use of obstetric services. 2nd ed. (1997). p. 1–103.

[16] Chokshi M Patil B Khanna R Neogi SB Sharma J Paul VK Health systems in India. J Perinatol. (2016) 36(3):S9–S12. 10.1038/jp.2016.184 27924110 PMC5144115

[17] Ministry of Health and Family Welfare Government of India. National Urban Health Mission: Framework for Implementation. (2013). Available from: https://nhm.gov.in/images/pdf/NUHM/Implementation_Framework_NUHM.pdf

[18] Chaturvedi S Randive B Diwan V Costsa AD. Quality of obstetric referral services in India’s JSY cash transfer programme for institutional births: a study from madhya pradesh province. PLoS ONE. (2014) 9(5):e96773. 10.1371/journal.pone.0096773 24810416 PMC4014551

[19] Give C Ndima S Steege R Ormel H McCollum R Theobald S Strengthening referral systems in community health programs: A qualitative study in two rural districts of Maputo Province, Mozambique. BMC Health Serv Res (2019) 19(1):1–11. 31035983 10.1186/s12913-019-4076-3 PMC6489304

[20] Daniels AA Abuosi A. Improving emergency obstetric referral systems in low and middle income countries: a qualitative study in a tertiary health facility in Ghana. BMC Health Serv Res. (2020) 20(1):1–10. 10.1186/s12913-020-4886-3 PMC6954606 31924203

[21] Aggarwal N Singla R Dhaliwal L Suri V. Audit of emergency obstetric referrals—A pilot study from tertiary care centre of north India. Bangladesh J Obstet Gynaecol. (2015) 30(1):25–9. 10.3329/bjog.v30i1.30504

[22] Akande TM. Referral system in Nigeria: study of a tertiary health facility. Ann Afr Med. (2004) 3(3):130–3.

[23] Ohara K Meléndez V Uehara N Ohi G. Study of a patient referral system in the republic of Honduras. Health Policy Plan. (1998) 13(4):433–45. 10.1093/heapol/13.4.433 10346035

[24] Siddiqi S Kielmann AA Ali N Ghaffar A Mumtaz Z. The effectiveness of patient referral in Pakistan. Health Policy Plan. (2001) 16(2):193–8. 10.1093/heapol/16.2.193 11358921

[25] Bhattacharyya S Srivastava A Saxena M Gogoi M Dwivedi P Giessler K. Do women’s perspectives of quality of care during childbirth match with those of providers? A qualitative study in Uttar Pradesh, India. Glob Health Action. (2018) 11(1):1527971. 10.1080/16549716.2018.1527971 30295161 PMC6179056

[26] Office of the Registrar General and Census Commissioner. Government of India; Ministry of Home Affairs. Census of India 2011. Available from: https://censusindia.gov.in/census.website/

[27] Strand RT de Campos PA Paulsson G de Oliveira J Bergström S. Audit of referral of obstetric emergencies in Angola: a tool for assessing quality of care. Afr J Reprod Health. (2009) 13(2):75–85. 20690251

[28] Kant S Kaur R Malhotra S Haldar P Goel AD. Audit of emergency obstetric referrals from a secondary level hospital in Haryana, North India. J Family Med Prim Care. (2018) 7:137–41. 10.4103/jfmpc.jfmpc_348_17 29915747 PMC5958555

[29] Rohit G P Nitin C S Vasantrao G N Professor A. Maternal and fetal outcome in referred patients to tertiary care center. Scholars J Appl Med Sci. (2016) 4(5C):1624–31.

[30] Sabale U Patankar AM. Study of maternal and perinatal outcome in referred obstetrics cases. J Evol Med Dent Sci. (2015) 4(26):4448–55. 10.14260/jemds/2015/643

[31] Prathiba P Niranjjan R Maurya DK Lakshminarayanan S. Referral chain of patients with obstetric emergency from primary care to tertiary care: a gap analysis. J Family Med Prim Care. (2020) 9(1):347. 10.4103/jfmpc.jfmpc_836_19 32110617 PMC7014899

[32] Pembe AB Carlstedt A Urassa DP Lindmark G Nyström L Darj E. Effectiveness of maternal referral system in a rural setting: a case study from Rufiji district, Tanzania. BMC Health Serv Res. (2010) 10(1):1–9. 10.1186/1472-6963-10-326 21129178 PMC3003655

[33] Jahn A Iang MD Shah U Diesfeld HJ. Maternity care in rural Nepal: a health service analysis. Trop Med Int Health. (2000) 5(9):657–65. 10.1046/j.1365-3156.2000.00611.x 11044281

[34] Dutta I Priyanka R Dasgupta S Khan M Saha P. Obstetrics referrals: maternal and perinatal outcome in medical college hospital in eastern India. Indian J Obstet Gynecol Res. (2020) 7(1):91–9. 10.18231/j.ijogr.2020.019

[35] Nagavarapu S Shridhar V Kropp N Murali L Swathi S Balachandra RP Reasons for obstetric referrals from community facilities to a tertiary obstetric facility: a study from Southern Karnataka. J Family Med Prim Care. (2019) 8(7):2378–83. 10.4103/jfmpc.jfmpc_308_19 31463261 PMC6691466

[36] Assefa EM Berhane Y. Delays in emergency obstetric referrals in Addis Ababa hospitals in Ethiopia: a facility-based, cross-sectional study. BMJ Open. (2020) 10(6):e033771. 10.1136/bmjopen-2019-033771 32580981 PMC7312330

[37] Bharati K Kumar B Bharati S Author C. Obstetric referral pattern in a tertiary care centre: a prospective observational study. Eur J Mol Clin Med. (2020) 07(10):4024–30.

[38] Ghardallou M Limam M Khelifi A Khairi O Khairi H Mtiraoui A Obstetric referrals to a tertiary care maternity: a descriptive study. Pan Afr Med J. (2019) 33:306. 10.11604/pamj.2019.33.306.16906 31692846 PMC6815507

[39] Ekka A Gupta SA Sahu D Minj AM Soni GP. Assessment of infrastructure of first referral unit facilities in Surguja division: a responsibility of providing emergency obstetric care. Int J of Commun Med Public Health. (2019) 6(12):5168. 10.18203/2394-6040.ijcmph20195464

[40] Anwar I Kalim N Koblinsky M. Quality of obstetric care in public-sector facilities and constraints to implementing emergency obstetric care services: evidence from high- and low-performing districts of Bangladesh. J health. Popul Nutr. (2009) 27(2):139–55. 10.3329/jhpn.v27i2.3327 19489412 PMC2761772

[41] Geleto A Chojenta C Musa A Loxton D. Barriers to access and utilization of emergency obstetric care at health facilities in sub-Saharan Africa: a systematic review of literature. Syst Rev. (2018) 7(1):1–14. 10.1186/s13643-017-0670-9 30424808 PMC6234634

[42] Knight HE Self A Kennedy SH. Why are women dying when they reach hospital on time? A systematic review of the “third delay.”. PLoS One. (2013) 8(5):e63846. 10.1371/journal.pone.0063846 23704943 PMC3660500

[43] Raman PS Sharma B Mavalankar D Upadhyaya M. Assessing the Regional and District Capacity for Operationalizing Emergency Obstetric Care through First Referral Units in Gujarat. (2009).

[44] Akbari A Mayhew A Al-Alawi MA Grimshaw J Winkens R Glidewell E Interventions to improve outpatient referrals from primary care to secondary care. Cochrane Database Syst Rev. (2008) 4:CD005471. 10.1002/14651858.CD005471 PMC4164370 18843691

[45] Ameyaw EK Amoah RM Njue C Tran NT Dawson A. Audit of documentation accompanying referred maternity cases to a referral hospital in northern Ghana: a mixed-methods study. BMC Health Serv Res. (2022) 22(1):4–11. 10.1186/s12913-022-07760-6 35296312 PMC8925182

[46] Murray SF Pearson SC. Maternity referral systems in developing countries: current knowledge and future research needs. Soc Sci Med. (2006) 62(9):2205–15. 10.1016/j.socscimed.2005.10.025 16330139

[47] Ameyaw EK Njue C Tran NT Dawson A. Quality and women’s satisfaction with maternal referral practices in sub-Saharan African low and lower-middle income countries: a systematic review. BMC Pregnancy Childbirth. (2020) 20(1):1–16. 10.1186/s12884-020-03339-3 PMC7656726 33176732

[48] Harahap NC Handayani PW Hidayanto AN. Barriers and technologies of maternal and neonatal referral system in developing countries: a narrative review. Inf Med Unlocked. (2019) 15:100184. 10.1016/j.imu.2019.100184

[49] World Health Organization. WHO Statement on caesarean section rates. Geneva: World Health Organization (2015).

[50] Betrán AP Torloni MR Zhang JJ Gülmezoglu AM Aleem HA Althabe F WHO Statement on caesarean section rates. BJOG. (2016) 123(5):667–70. 10.1111/1471-0528.13526 26681211 PMC5034743

[51] Sørbye IK Vangen S Oneko O Sundby J Bergsjø P. Caesarean section among referred and self-referred birthing women: a cohort study from a tertiary hospital, northeastern Tanzania. BMC Pregnancy Childbirth. (2011) 11:1–10. 10.1186/1471-2393-11-55 21798016 PMC3160415

